# Incidence of acute cerebral infarction or space occupying lesion among patients with isolated dizziness and the role of D-dimer

**DOI:** 10.1371/journal.pone.0214661

**Published:** 2019-03-28

**Authors:** Sion Jo, Taeoh Jeong, Jae Baek Lee, Youngho Jin, Jaechol Yoon, Boyoung Park

**Affiliations:** 1 Department of Emergency Medicine, Research Institute of Clinical Medicine of Chonbuk National University and Biomedical Research Institute of Chonbuk National University Hospital, Jeonju-si, Korea; 2 College of Medicine, Department of Medicine, Hanyang University, Seoul, Republic of Korea; University at Buffalo, UNITED STATES

## Abstract

**Background:**

To determine the incidence of acute cerebral infarction or space occupying lesion (SOL) among patients with isolated vertigo or dizziness (IVD) and to evaluate the role of cerebellar function test (CFT) and D-dimer to discriminate ACI/SOL and non-ACI/SOL.

**Methods:**

A retrospective study of consecutive emergency department (ED) patients with IVD during one year was conducted. ACI was based on the diffusion-weighted magnetic resonance imaging (DW-MRI), and SOL was based on the concurrent MRI sequences. A sensitivity analysis of CFT and D-dimer was also performed.

**Results:**

Among the 468 patients enrolled, 13 patients (2.8%) had ACI, 11 at cerebellum, 1 at occipital lobe, and 1 at centrum semiovale. Twenty-five patients (5.3%) had SOL. Aneurysm is most frequent (n = 7), followed by meningioma (n = 4) and venous anomaly (n = 4). In total, ACI/SOL was found in 8.1% (n = 38). Abnormal findings in finger-to-nose (FN), heel-to-shin (HTS), and rapid alternative movement (RAM) tests were significantly higher in ACI or ACI/SOL group, while gait disturbance, tandem gait abnormality, and Romberg’s test were not. CFT sensitivities were low for ACI as well as for ACI/SOL, but specificities were high for ACI and ACI/SOL. D-dimer level showed a sensitivity of 100% at >0.18 mg/L for ACI and >0.15 mg/L for ACI/SOL. However, specificity was low at corresponding D-dimer level. Among the subgroup (n = 411) who did not show any abnormality in CFT, 9 patients (2.2%) had ACI, and 33 patients (8.0%) had ACI/SOL.

**Conclusion:**

The present study reports a clinically significant incidence of ACI/SOL among ED patients with IVD. D-dimer showed high sensitive and low specificity, while CFT showed low sensitivity and high specificity.

## Introduction

In the United States alone, an estimated 7.5 million patients with dizziness are seen in ambulatory care setting [1]. When patients complain of dizziness, the term can refer to a broad range of symptoms including vertigo, lightheadedness, presyncope, faintness, gait instability, or even drowsiness due to an infectious disease. Likewise, various diseases may also cause dizziness including vestibular neuronitis, benign paroxysmal positional vertigo (BPPV), Meniere’s disease, among others. From potential diagnoses, stroke is a major concern that should be ruled out during initial assessment, because it is critical and life-threatening if misdiagnosed [2]. Stroke is difficult to distinguish from other diseases if a patient presents symptoms of dizziness without any accompanying focal neurological deficits, which traditionally refers to isolated vertigo or dizziness (IVD). A small infarction involving the cerebellum or brain stem may present without other neurologic symptoms or signs [3]. Furthermore, diagnosis becomes even more challenging if features of stroke and peripheral-type dizziness are combined [4]. Such complexity often calls for scrupulous evaluation, including brain imaging, to confirm whether it is stroke or not.

As an expensive test is not the first diagnostic modality, prior tests are frequently required. When evaluating patients with IVD, some findings from neurologic examination or red-flag signs clinically guide the decision to require a brain MRI. Abnormal findings on neurologic examination, particularly cerebellar function test (CFT), are often questionable in real practice. Some factors, such as senile change or nystagmus, can affect the cerebellar function, which coordinates fine muscle tone or balance. Additionally, patients with vestibular neuronitis or BPPV generally find it difficult to walk in the acute phase and even barely open their eyes, sequentially making them unable to undergo CFT test. Thus, clinically, IVD is often regarded as dizziness without a neurologic deficit—except CFT results—although IVD traditionally and strictly means dizziness without any neurologic deficit including CFT. Moreover, brain imaging can reveal space occupying lesion (SOL), such as a tumor or a venous anomaly, as well as acute cerebral infarction (ACI). However, previous studies related with IVD did not show any specific concern regarding this issue [4–7].

This study sought to determine the incidence of ACI or SOL among IVD patients, defined as dizziness patients without any neurologic deficit except CFT findings. Also, the clinical role of CFT for ACI or SOL prediction was examined, including finger-to-nose (FN), heel-to-shin (HTS), rapid alternative motion (RAM), gait disturbance, tandem gait test, and Romberg’s test. The initial serum D-dimer level, one of the routine laboratory tests in the study hospital’s emergency department (ED), was also included. It was evaluated along with CFT for the potential role among patients with IVD, taking into consideration its current practical role in thromboembolic diseases [8–10].

## Materials and methods

### Study design and subjects

A retrospective chart review study was performed, which was approved by the institutional review board (IRB) of the Chonbuk National University Hospital (CUH 2018-08-022). The IRB waived the requirement for informed consent for all subjects in the present study. We followed the ethical standards of the Helsinki Declaration of 1975, as revised in 2000. The study hospital is a 1,200-bed urban—academic, tertiary care university hospital. The Standards for the Reporting of Diagnostic Accuracy (STARD) recommendations was used as reference in analyzing the results [11–12].

Adult (> or = 18 years) ED patients who registered a chief complaint of dizziness or vertigo within the one-year study period (January 1, 2015 –December 31, 2015) were screened for eligibility. In the present study, IVD is defined as dizziness without any altered mentality, confusion, diplopia, aphasia, dysarthria, cranial nerve palsy—including facial palsy, motor, or sensory deficit—and without any other mechanisms such as current infection, metabolic derangements, recent trauma, anaphylaxis, drug, and other similar means. According to the definition of IVD, the abnormal CFT results (FN, HTS, RAM, gait disturbance, tandem gait, and Romberg’s test) were not regarded as exclusion criteria. Exclusion criteria included the following: 1) patients with other concurrent symptoms or mechanisms; 2) patients with underlying malignancy, aneurysm, or were transferred during recent infarction treatment; and 3) patients who did not check MRI. The second exclusion criterion was included to reduce the bias.

**Fig 1:**
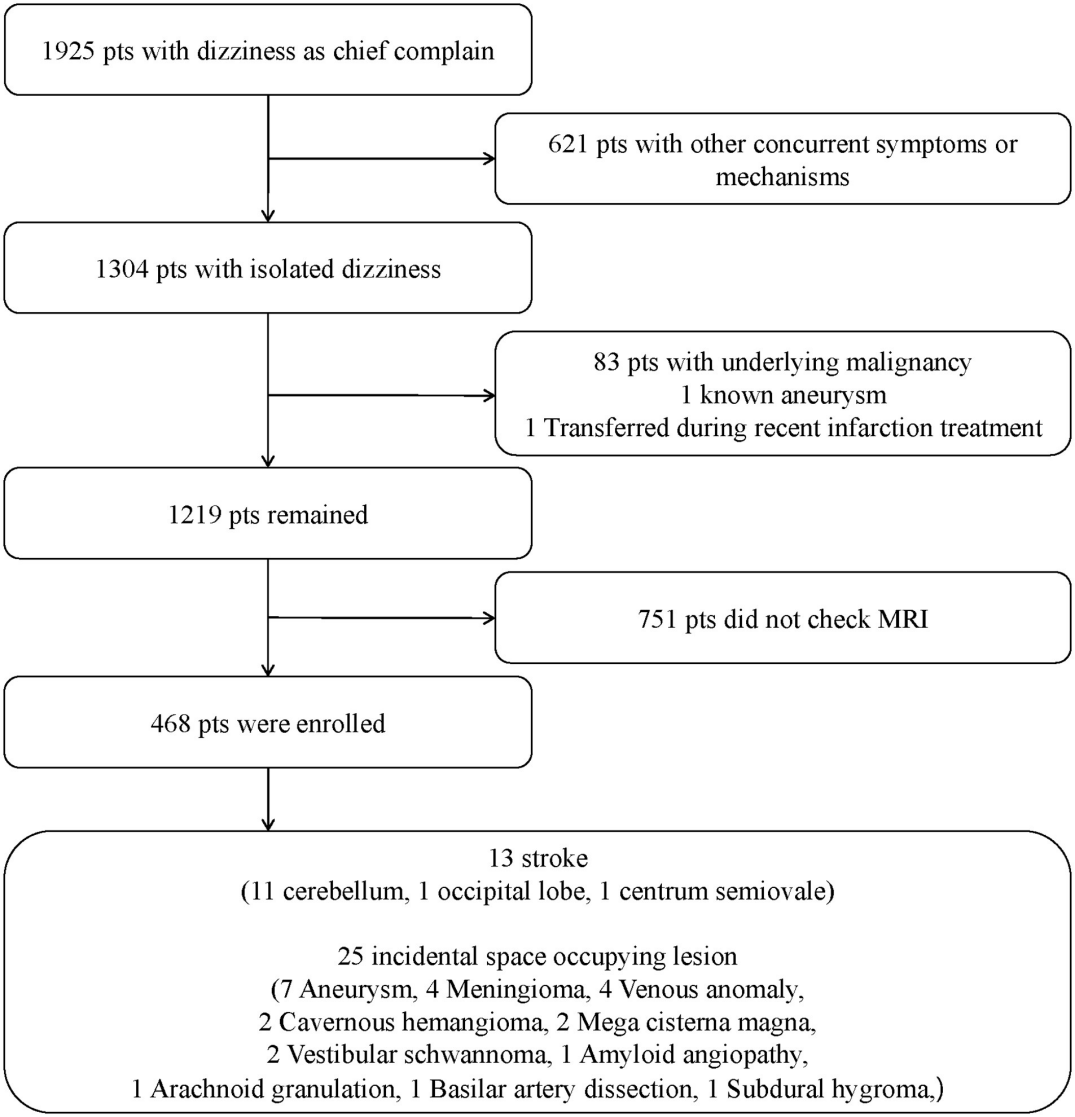
Study flow.

A total of 1,925 ED patients were screened, and the 621 patients who did not meet the IVD definition were excluded from the enrollment. Patients with underlying malignancy (n = 83) and known aneurysm (n = 1), and were transferred during recent infarction treatment (n = 1) were excluded. Among the remaining patients, 751 underwent MRI and were thus excluded from the analysis. As a result, a total of 468 patients were enrolled in the present study (Fig 1). Among 468 enrolled patients, 451 patients (96.4%) underwent magnetic resonance angiography (MRA) sequence along with DW-MRI.

### Outcome measures

The primary outcome was the number of ACI based on the DW-MRI findings. The secondary outcome was the sum of ACI and SOL (ACI/SOL), found through the other sequences which were performed along with DW sequence, such as T1, T2, Fluid-attenuated inversion recovery (FLAIR), and MRA sequence.

### Data collection and processing

Demographics, clinical data, physical findings at ED presentation, initial laboratory results (within two hours of ED arrival), and MRI results (within 48 hours of ED arrival) were collected by a trained abstractor according to the guidelines recommended by Gilbert et al [13]. These included the following: age; sex; emergency medical system (EMS) used; comorbidities such as hypertension, diabetes mellitus, dyslipidemia, cerebrovascular disease, malignancy, and aneurysm; initial vital signs; national early warning score (NEWS); assumed peripheral origin at the ED; and whether to admit to the intensive care unit (ICU) or ward. Aside from these, the following information was also collected: spinning/whirling feature; positional aggravation feature; results of FN, HTS, RAM, gait disturbance, tandem gait, and Romberg’s test; any type of nystagmus; and D-dimer level. CFT results were based on the chart written by junior neurology residents, who circulated the ED as part of training. MRI results were based on the report by the board-certified radiologists who specialized in neuroradiology. The normal range of D-dimer was below 0.50 mg/L in the study hospital.

### Statistical analysis

All continuous data were presented as the mean and standard deviation (SD) for normally distributed data, the median and interquartile range (IQR) for non-normally distributed data, and discrete data were presented as both the count and the percentage. Results of logistic regression analyses were presented as the odds ratio with a 95% confidence interval. Statistical significance was defined as a two-sided p<0.05.

Comparison of normally distributed data was performed using an independent sample t-test. For non-normally distributed data, comparisons were performed using the Mann-Whitney U test or Kruskal—Wallis test. For categorical data, the chi-square test (with a Fisher’s exact test, if necessary) for 2×2 tables was used. Results were considered significant at a threshold of p<0.05 (two-tailed). Associations between the presence of primary/secondary outcomes and each potential variable were first quantified using univariate logistic regression analyses. Regression results are expressed as ORs with a 95% confidence interval (CI).

All analyses were performed using Stata 11.1 (StataCorp LP, TX, USA) and SAS 9.1 (SAS Institute Inc., Cary, NC, USA).

## Results

### Characteristics of study subjects

Table 1 summarizes the baseline characteristics of the enrolled subjects and unadjusted odd ratios of collected variables. The mean age was 60.8±14.0 years, and 204 (43.6%) patients were male. Hypertension was the most frequent comorbidity (40.8%), followed by DM (16.7%), and dyslipidemia / cerebrovascular disease (13.7%, respectively). Vital signs were within normal range. Two (0.4%) patients were admitted to the ICU, and 121 (25.9%) patients were admitted to the ward. Spinning or whirling feature was seen in 311 (66.5%) patients, and positional aggravation feature was seen in 339 (72.4%) patients. FN, HTS, and RAM were tested in most of the patients, while Romberg’s test was performed in 182 patients (38.9%). A slight number of patients (n = 11, 2.6%) showed abnormal findings in the FN, HTS, and RAM tests. Forty-three (12.9%) patients showed gait disturbance, and 49 (15.0%) patients showed tandem gait abnormality. Thirteen (7.1%) patients showed positive findings in Romberg’s test, and 213 (46.6%) patients showed some type of nystagmus. The mean D-dimer was 0.5±0.8 mg/L.

**Table 1:** Baseline characteristics of enrolled isolated vertigo or dizziness patients.

Variable	All	No-ACI	ACI	No-ACI or SOL	ACI or SOL
Number, n (%)	468 (100)	455 (97.2)	13 (2.8)	430 (91.9)	38 (8.1)
Age, year	60.8±14.0	60.0 [51.0;72.0]	66.5 [62.0;74.0]a	60.0 [51.0;72.0]	66.0 [56.0;74.0]a
Age <65, n (%)	274 (58.6)	271 (59.6)	3 (23.1)a	257 (59.8)	17 (44.7)
Age 65–74	107 (22.8)	99 (21.8)	8 (61.5)a	95 (22.1)	12 (31.6)
Age ≥75	87 (18.6)	85 (18.7)	2 (15.4)a	78 (18.1)	9 (23.7)
Male	204 (43.6)	196(43.1)	8 (61.5)	186 (43.3)	18 (47.4)
EMS use	165 (35.3)	159 (34.9)	6 (46.2)	146 (34.0)	19 (50.0)
Comorbidity, n (%)					
Hypertension	191 (40.8)	186 (40.9)	5 (38.5)	175 (40.7)	16 (42.1)
Diabetes mellitus	78 (16.7)	76 (16.7)	2 (15.4)	74 (17.2)	4 (10.5)
Dyslipidemia	64 (13.7)	63 (13.9)	1 (7.7)	59 (13.7)	5 (13.2)
Cerebrovascular disease	64 (13.7)	61 (13.4)	3 (23.1)	57 (13.3)	7 (18.4)
Physiology					
SBP, mmHg	139.6±22.0	140.0 [123.0;150.0]	140.0 [140.0;150.0]	140.0 [120.0;150.0]	140.0 [130.0;150.0]
DBP, mmHg	83.8±12.9	80.0 [80.0;90.0]	90.0 [80.0;100.0]	80.0 [80.0;90.0]	90.0 [80.0;90.0]
PR, bpm	78.2±33.0	76.0 [70.0;84.0]	84.0 [72.0;88.0]	76.0 [71.0;84.0]	79.0 [68.0;88.0]
RR, bpm	19.1±7.9	18.0 [18.0;20.0]	18.0 [18.0;19.0]	18.0 [18.0;20.0]	18.0 [18.0;19.0]
BT, °C	36.3±0.4	36.4 [36.1;36.6]	36.3 [36.2;36.4]	36.3 [36.0;36.6]	36.4 [36.2;36.5]
NEWS	0.8±1.0	0.0 [0.0;1.0]	0.0 [0.0;1.0]	0.0 [0.0;1.0]	0.0 [0.0;1.0]
Peripheral origin	277 (59.2)	275 (60.4)	2 (15.4)a	258 (60.0)	19 (50.0)
ICU admission	2 (0.4)	0	2 (15.4)a	0	2 (5.3)a
Ward admission	121 (25.9)	114 (25.1)	7 (53.8)a	105 (24.4)	16 (42.1)a
Dizziness feature, n (%)					
Spinning/whirling	311 (66.5)	304 (66.8)	7 (53.8)	288 (67.0)	23 (60.5)
Positional aggravation	339 (72.4)	330 (72.5)	9 (69.2)	309 (71.9)	30 (78.9)
Any nystagmus (457)	213 (46.6)	209 (47.0)	4 (33.3)	197 (46.9)	16 (43.2)
CFT abnormality, n(%)					
FN (n = 421)	10 (2.4)	7 (1.7)	3 (23.1)a	7 (1.8)	3 (8.1)a
HTS (n = 421)	10 (2.4)	7 (1.7)	3 (23.1)a	7 (1.8)	3 (8.1)a
RAM (n = 420)	10 (2.4)	7 (1.7)	3 (23.1)a	7 (1.8)	3 (8.1)a
Gait (n = 334)	43 (12.9)	41 (12.6)	2 (22.2)	41 (13.2)	2 (8.3)
Tandem gait (n = 327)	49 (15.0)	47 (14.7)	2 (25.0)	47 (15.4)	2 (9.1)
Romberg positive (n = 182)	13 (7.1)	12 (6.7)	1 (33.3)	11 (6.4)	2 (18.2)
D-dimer, mg/L (n = 347)	0.5±0.8	0.5±0.8	0.9±0.9	0.5±0.8	0.9±1.3a

Abbreviations: ACI = acute cerebral infarction; EMS = emergency medical service; SBP = systolic blood pressure; DBP = diastolic blood pressure; PR = pulse rate; RR = respiratory rate; BT = body temperature; NEWS = national early warning score; ICU = intensive care unit; CFT = cerebellar function test; FN = finger to nose; HTS = heel to shin; RAM = rapid alternative motion. Peripheral origin means that the assumed peripheral origin is at ED. ‘a’ denotes a statistical significance of p-value < 0.05

ACI, based on the DW-MRI findings, was found in 13 (2.8%) patients. Patients with ACI were older than patients with non-ACI. However, sex, comorbidities, and physiologic variables did not show significant differences. More patients with ACI were admitted to the ICU and ward. No significant difference was found regarding spinning/whirling or positional aggravation feature. Abnormal findings in the FN, HTS, and RAM were higher in ACI patients than in non-ACI patients. There were no significant differences regarding gait disturbance, tandem gait abnormality, Romberg’s test, any nystagmus, and D-dimer level.

ACI/SOL was found in 38 (8.1%) patients, and they were older than patients who were not. Variables that showed significant differences between ACI/SOL patients and non-ACI/SOL were similar in cases of comparison between ACI and non-ACI patients. Exceptionally, the D-dimer level was significantly higher in ACI/SOL patients.

Cerebellar infarction was found in 11 patients, occipital lobe infarction in 1 patient, and centrum semiovale infarction in 1 patient. Four out of the 11 patients with cerebellar infarction showed very tiny and spot infarctions, and no significant abnormal findings were noted in their CFT. SOL was found in 25 patients (Fig 1). Most common findings were aneurysm (n = 7, 1 at anterior communicating artery, 1 at posterior communicating artery, 1 at middle cerebral artery bifurcation, 1 at superior cerebellar artery orifice, 1 at basilar tip, and 2 at distal internal carotid artery), followed by meningioma (n = 4, 1 at frontal lobe, 1 at midline fax frontal area, 1 at occipital convexity, and 1 at frontal base & parietal lobe) and venous anomaly (n = 4, 2 at cerebellar area, 1 at frontal lobe, and 1 at frontal & cerebellar area). The remains were Cavernous hemangioma (n = 2, 1 at parietal lobe, and 1 at temporal medial portion), mega cisterna magna (n = 2), vestibular schwanoma (n = 2, 2 at internal auditory canal), amyloid angiopathy (n = 1, at both lobe), arachnoid granulation (n = 1, at transversre venous sinus), basilar artery dissection (n = 1, at mid portion), and subdural hygroma (n = 1, at both frontal area).

**Fig 2:**
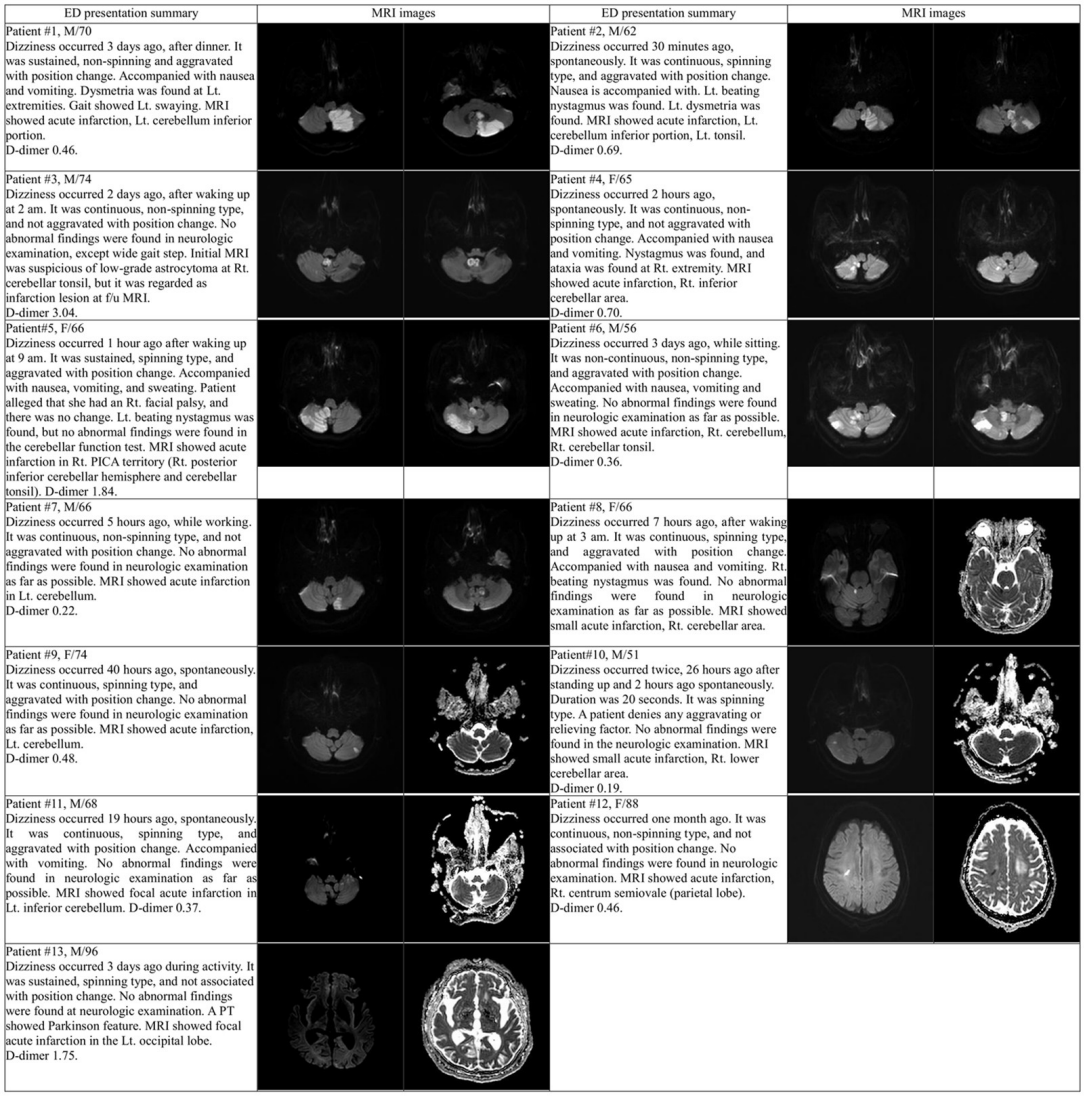
Case summary of isolated dizziness patients diagnosed with acute cerebral infarction based on DW-MRI sequence.

Clinical symptoms and MRI findings of 13 ACI patients were summarized in Fig 2. Among those, 4 patients (patient numbers 1–4) showed abnormal findings on CFT. The infarction size in the MRI of 6 patients (patient numbers 8–13) was found to be subtle.

Diagnostic performance was evaluated and results are found in Table 2. FN, HTS, RAM, gait disturbance, tandem gait, and Romberg’s test showed low sensitivity, high specificity, and low PPV for ACI as well as ACI/SOL. LR+ of FN, HTS, and RAM was high (13.4–13.5) for ACI. NPV for ACI showed high value, but it was not enough considering that the prevalence is very low (2.8%). The SN and PPV of gait disturbance, tandem gait, and Romberg’s test were also low. No clinically notable findings were found regarding spinning/whirling, positional aggravation feature, and nystagmus. Interestingly, the discriminative value of D-dimer was greatest among the collected variables (0.70 for ACI and 0.64 for ACI/SOL). The D-dimer value that showed 100% SN and NPV was >0.18 mg/L for ACI, and >0.15 mg/L for ACI/SOL. Of note, only a D-dimer difference of 0.01 mg/L showed a substantial decrement in SN for both ACI patients and ACI/SOL patients. D-dimer showed low specificity at corresponding D-dimer level, which raised a concern of high false positive when physicians relied on the D-dimer level only.

**Table 2:** Characteristics of cerebellar function test and D-dimer level for the prediction of acute cerebral infarction or space occupying lesions among patients with isolated vertigo or dizziness.

Variable	Outcome	UOR (95% CI)	AUROC (95% CI)	Cutoff level	SN,%(95% CI)	SP,%(95% CI)	PPV,%(95% CI)	NPV,%(95% CI)	LR+, (95% CI)	LR-, (95% CI)
FN	ACI	17.2 (3.9–76.3)	0.61 (0.49–0.73)	Abnormal	23.1 (5.0–53.8)	98.3 (96.5–99.3)	30.0 (6.7–65.2)	97.6 (95.6–98.8)	13.5 (3.9–46.2)	0.8 (0.6–1.1)
	ACI or SOL	4.8 (1.2–19.2)	0.53 (0.49–0.58)	Abnormal	8.1 (1.7–21.9)	98.2 (96.3–99.3)	30.0 (6.7–65.2)	91.7 (88.6–94.2)	4.5 (1.2–16.5)	0.9 (0.9–1.0)
HTS	ACI	17.2 (3.9–76.3)	0.61 (0.49–0.73)	Abnormal	23.1 (5.0–53.8)	98.3 (96.5–99.3)	30.0 (6.7–65.2)	97.6 (95.6–98.8)	13.5 (3.9–46.2)	0.8 (0.6–1.1)
	ACI or SOL	4.8 (1.2–19.2)	0.53 (0.49–0.58)	Abnormal	8.1 (1.7–21.9)	98.2 (96.3–99.3)	30.0 (6.7–65.2)	91.7 (88.6–94.2)	4.5 (1.2–16.5)	0.9 (0.9–1.0)
RAM	ACI	17.2 (3.9–76.1)	0.61 (0.49–0.73)	Abnormal	23.1 (5.0–53.8)	98.3 (96.5–99.3)	30.0 (6.7–65.2)	97.6 (95.6–98.8)	13.4 (3.9–46.1)	0.8 (0.6–1.1)
	ACI or SOL	4.7 (1.2–19.2)	0.53 (0.49–0.58)	Abnormal	8.1 (1.7–21.9)	98.2 (96.3–99.3)	30.0 (6.7–65.2)	91.7 (88.6–94.2)	4.4 (1.2–16.4)	0.9 (0.9–1.0)
Gait	ACI	2.0 (0.4–9.9)	0.55 (0.40–0.69)	Abnormal	22.2 (2.8–60.0)	87.4 (83.3–90.8)	4.7 (0.6–15.8)	97.6 (95.1–99.0)	1.8 (0.5–6.2)	0.9 (0.6–1.3)
	ACI or SOL	0.6 (0.1–2.6)	0.48 (0.42–0.54)	Abnormal	8.3 (1.0–27.0)	86.8 (82.5–90.3)	4.7 (0.6–15.8)	92.4 (88.8–95.2)	0.6 (0.2–2.5)	1.1 (0.9–1.2)
Tandem gait	ACI	1.9 (0.4–9.8)	0.55 (0.39–0.71)	Abnormal	25.0 (3.2–65.1)	85.3 (80.9–89.0)	4.1 (0.5–14.0)	97.8 (95.4–99.2)	1.7 (0.5–5.8)	0.9 (0.6–1.3)
	ACI or SOL	0.5 (0.1–2.4)	0.47 (0.40–0.53)	Abnormal	9.1 (1.1–29.2)	84.6 (80.0–88.5)	4.1 (0.5–14.0)	92.8 (89.1–95.6)	0.6 (0.1–2.3)	1.1 (0.9–1.2)
Romberg	ACI	7.0 (0.6–82.3)	0.63 (0.31–0.96)	Positive	33.3 (0.8–90.6)	93.3 (88.6–96.5)	7.7 (0.2–36.0)	98.8 (95.8–99.9)	5.0 (0.9–27.0)	0.7 (0.3–1.6)
	ACI or SOL	3.2 (0.6–16.8)	0.56 (0.44–0.68)	Positive	18.2 (2.3–51.8)	93.6 (88.8–96.7)	15.4 (1.9–45.4)	94.7 (90.1–97.5)	2.8 (0.7–11.2)	0.9 (0.7–1.2)
Spinning/whirling	ACI	0.6 (0.2–1.8)	0.44 (0.29–0.58)	Existence	53.8 (25.1–80.8)	33.2 (28.9–37.7)	2.3 (0.9–4.6)	96.2 (91.9–98.6)	0.8 (0.5–1.3)	1.4 (0.8–2.5)
	ACI or SOL	0.8 (0.4–1.5)	0.47 (0.39–0.55)	Existence	60.5 (43.4–76.0)	33.0 (28.6–37.7)	7.4 (4.8–10.9)	90.4 (84.7–94.6)	0.9 (0.7–1.2)	1.2 (0.8–1.9)
Positional aggravation	ACI	0.9 (0.3–2.8)	0.48 (0.35–0.62)	Existence	69.2 (38.6–90.9)	27.5 (23.4–31.8)	2.7 (1.2–5.0)	96.9 (92.3–99.1)	1.0 (0.7–1.4)	1.1 (0.5–2.6)
	ACI or SOL	1.5 (0.7–3.3)	0.54 (0.47–0.60)	Existence	78.9 (62.7–90.4)	28.1 (23.9–32.6)	8.9 (6.1–12.4)	93.8 (88.1–97.3)	1.1 (0.9–1.3)	0.7 (0.4–1.4)
Any nystagmus	ACI	0.6 (0.2–1.9)	0.43 (0.29–0.57)	Existence	33.3 (9.9–65.1)	53.0 (48.3–57.7)	1.9 (0.5–4.7)	96.7 (93.6–98.6)	0.7 (0.3–1.6)	1.3 (0.8–1.9)
	ACI or SOL	0.9 (0.4–1.7)	0.48 (0.40–0.57)	Existence	43.2 (27.1–60.5)	53.1 (48.2–58.0)	7.5 (4.4–11.9)	91.4 (87.1–94.6)	0.9 (0.6–1.4)	1.1 (0.8–1.4)
D-dimer	ACI	1.3 (0.9–2.0)	0.70 (0.56–0.84)							
		Omitted		>0.18 > = 0.19, mg/L	100 (73.5–100)	23.0 (18.6–27.9)	4.4 (2.3–7.6)	100 (95.3–100)	1.3 (1.2–1.4)	0
		3.7 (0.5–28.9)		>0.19 > = 0.20	91.7 (61.5–99.8)	25.1 (20.5–30.1)	4.2 (2.1–7.4)	98.8 (93.6–100)	1.2 (1.0–1.5)	0.3 (0.1–2.2)
		4.4 (0.6–34.2)		>0.21 > = 0.22	91.7 (61.5–99.8)	28.4 (23.6–33.5)	4.4 (0.7–1.9)	99.0 (94.3–100)	1.3 (1.1–1.5)	0.3 (0.0–1.9)
		2.2 (0.5–10.3)		>0.22 > = 0.23	83.3 (51.6–97.9)	30.7 (25.8–36.0)	4.1 (2.0–7.5)	98.1 (93.3–99.8)	1.2 (0.9–1.6)	0.5 (0.2–1.9)
	ACI or SOL	1.4 (1.1–1.9)	0.64 (0.53–0.75)							
		Omitted		>0.15 > = 0.16	100 (87.7–100)	14.1 (10.5–18.4)	9.3 (6.3–13.1)	100 (92.1–100)	1.2 (1.1–1.2)	0
		5.5 (0.7–41.4)		>0.16 > = 0.17	96.4 (81.7–99.9)	16.9 (13.0–21.5)	9.3 (6.2–13.2)	98.2 (90.3–100)	1.2 (1.1–1.3)	0.2 (0.0–1.5)
		2.3 (0.7–7.7)		>0.17 > = 0.18	89.3 (71.8–97.7)	21.3 (17.0–26.2)	9.1 (6.0–13.1)	95.8 (88.1–99.1)	1.1 (1.0–1.3)	0.5 (0.2–1.5)

Abbreviations: UOR = unadjusted odd ratio; AUROC = area of under the receiver operating characteristics; SN = sensitivity; SP = specificity; PPV = positive predictive value; NPV = negative predictive value; LR+ = likelihood ratio positive; LR- = likelihood ratio negative; CFT = cerebellar function test; FN = finger to nose; ACI = acute cerebral infarction; HTS = heel to shin; RAM = rapid alternative motion

Table 3 summarized the characteristics of IVD patients who did not show any abnormality on CFT. Among the 411 patients, the incidence of ACI was 2.2% and ACI/SOL was 8.0%, which is similar to those in the case of IVD patients (ACI 2.8% and ACI/SOL 8.1%). Other characteristics were also found to be similar. The D-dimer level was significantly higher in ACI/SOL group than in no-ACI/SOL group (1.0±1.4 mg/L vs. 0.5±0.8 mg/L).

**Table 3:** Baseline characteristics of enrolled isolated dizziness patients after excluding patients with abnormal cerebellar function test.

Variable	All	No-ACI	ACI	No-ACI or SOL	ACI or SOL
Number, n (%)	411 (100)	402 (97.8)	9 (2.2)	378 (92.0)	33 (8.0)
Age, year	60.6±13.9	60.0 [51.0;72.0]	67.0 [66.0;74.0]	60.0 [51.0;71.0]	66.0 [55.0;74.0]
Age <65, n (%)	240 (58.4)	238 (59.2)	2 (22.2)	225 (59.5)	15 (45.5)
Age 65–74	97 (23.6)	91 (22.6)	6 (66.7)	87 (23.0)	10 (30.3)
Age ≥75	74 (18.0)	73 (18.2)	1 (11.1)	66 (17.5)	8 (24.2)
Male	179 (43.6)	174 (43.3)	5 (55.6)	164 (43.4)	15 (45.5)
EMS use	145 (35.3)	142 (35.3)	3 (33.3)	129 (34.1)	16 (48.5)
Comorbidity, n (%)					
Hypertension	163 (39.7)	160 (39.8)	3 (33.3)	150 (39.7)	13 (39.4)
Diabetes mellitus	68 (16.6)	67 (16.7)	1 (11.1)	65 (17.2)	3 (9.1)
Dyslipidemia	54 (13.1)	54 (13.4)	0	51 (13.5)	3 (9.1)
Cerebrovascular disease	54 (13.1)	53 (13.2)	1 (11.1)	49 (13.0)	5 (15.2)
Physiology					
SBP, mmHg	139.3±21.8	140.0 [125.0;150.0]	140.0 [140.0;150.0]	140.0 [125.0;150.0]	140.0 [130.0;150.0]
DBP, mmHg	83.6±12.9	80.0 [80.0;90.0]	90.0 [80.0;100.0]	80.0 [80.0;90.0]	90.0 [80.0;90.0]
PR, bpm	78.2±34.9	76.0 [70.0;84.0]	78.0 [68.0;84.0]	76.0 [72.0;84.0]	76.0 [68.0;86.0]
RR, bpm	19.2±8.4	18.0 [18.0;20.0]	18.0 [18.0;19.0]	18.0 [18.0;20.0]	18.0 [18.0;19.0]
BT, °C	36.3±0.4	36.3 [36.1;36.5]	36.3 [36.2;36.4]	36.3 [36.1;36.5]	36.4 [36.2;36.5]
NEWS	0.8±1.0	0.0 [0.0;1.0]	0.0 [0.0;0.0]	0.0 [0.0;1.0]	0.0 [0.0;1.0]
Peripheral origin	248 (60.3)	246 (61.2)	2 (22.2)a	229 (60.6)	19 (57.6)
ICU admission	1 (0.2)	0	1 (11.1)a	0	1 (3.0)
Ward admission	102 (24.8)	97 (24.1)	5 (55.6)	88 (23.3)	14 (42.4)a
Dizziness feature, n (%)					
Spinning /Whirling	273 (66.4)	268 (66.7)	5 (55.6)	252 (66.7)	21 (63.6)
Positional aggravation	300 (73.0)	294 (73.1)	6 (66.7)	274 (72.5)	26 (78.8)
Any nystagmus (n = 400)	180 (45.0)	178 (45.4)	2 (25.0)	166 (45.1)	14 (43.8)
D-dimer, mg/L (n = 300)	0.5±0.9	0.5±0.9	0.9±1.0	0.5±0.8	1.0±1.4a

Abbreviations: ACI = acute cerebral infarction; EMS = emergency medical service; SBP = systolic blood pressure; DBP = diastolic blood pressure; PR = pulse rate; RR = respiratory rate; BT = body temperature; NEWS = national early warning score; ICU = intensive care unit. Peripheral origin means that the assumed peripheral origin is at ED. ‘a’ denotes a statistical significance of p-value < 0.05

## Discussion

In the present study, the incidence of ACI was 2.8% among IVD patients, and the incidence of ACI or SOL was 8.1%. CFTs showed low sensitivity but high positive likelihood ratio. It also showed high negative predictive value, but it seems to be limited in clinical use when the low incidence of outcome is taken into consideration. In the present cohort, extremely low D-dimer level showed 100% sensitivity and 100% negative predictive value, but low specificity.

Kerber et al. reported the prevalence of stroke among patients with isolated dizziness in their large population-based study, the Brain Attack Surveillance in Corpus Christi (BASIC) project [5]. Among the 1,666 patients with the principal presenting complaint of dizziness, 3.2% (n = 53) were validated to have a stroke or transient ischemic attack (TIA). A retrospective chart-based validation was conducted by neurologists to confirm these findings (dizziness in 23 cases, vertigo in 18 cases, imbalance in 11 cases, and more than one of these terms in one case). Among those 53 patients, only 9 patients were regarded as isolated dizziness. As 1,297 patients were regarded as isolated dizziness from the total number of patients, the prevalence of stroke or TIA was reported as 0.7% (9 of 1,297). They thus concluded that the proportion of stroke/TIA in patients with isolated dizziness symptom is low.

However, Doijiri et al. reported contrary results [6]. They enrolled 221 patients who were admitted due to sudden isolated vertigo or dizziness attack without other neurological symptoms, except for nystagmus, deafness, or tinnitus over 10 years. Brain computed tomography (CT) or MRI revealed recent stroke lesions in 25 patients (11.3%), (ischemic in 21 (9.5%) and hemorrhagic in 4 (1.8%) patients). In general, the lesions were small and localized in the cerebellum (n = 21), pons (n = 1), medulla oblongata (n = 1), or corona radiata (n = 1) [6].

Perloff et al. enrolled ED patients presenting with a chief complaint of dizziness or vertigo, without other symptoms or signs in narrative history or in their exam to suggest a central nervous system lesion. The patients’ workup included a brain MRI within 48 hours [7]. Among 136 patients, 5 patients (3.7%) showed acute cerebellar stroke based on DW-MRI sequence [7].

The historical flow of clinical perception for IVD can be estimated through previous studies. Under the condition of limited brain imaging, traditionally, ACI proportion was thought to be extremely low (0.7%) if there is no neurologic deficit among patients with dizziness. This was the beginning of the term “isolated dizziness.” However, after brain imaging was performed in certain and selected isolated dizziness patients, ACI prevalence was reported as high (9.5%), alarming the clinical practice for isolated dizziness. Taking into consideration Doijiri et al.’s study, the mean age of the cohort was nearly 70 (in the present study, it was 60). Therefore, the certain condition would be age, although age is not the only factor for brain MRI selection [6]. Thereafter, as MRI of the brain expanded among isolated dizziness, ACI prevalence reported a lower but clinically important proportion—approximately 2–3% as shown in the studies of Perloff et al [7]. A similar incidence was shown in the present study.

Compared to previous studies, the present study is more meaningful because it reported the prevalence of other clinically important findings—SOL and ACI. SOL was found among 25 patients, and the incidence of SOL is nearly double than ACI. Substantial cases of SOL suggested the chance of definite treatment options, such as stent insertion for aneurysm/dissection, or medical/surgical treatment for meningioma or schwannoma, although there was no follow up on the course of SOL patients. Furthermore, the present study proposed that an IVD would be a first presenting symptom among some SOL patients. To the knowledge of the researchers, it is the first report that estimates the incidence of SOL among IVD patients.

The other unique point of the present study is that the diagnostic performance of CFT was evaluated among IVD patients. Characteristically, CFT showed low sensitivity, meaning high false negative. It means that many ACI cases could be missed when CFT is used as the only basis in IVD patients. Therefore, CFT may not be a suitable screening test for the next evaluation step such as brain imaging. Negative predictive value seems to be high, but it seems to be limited in clinical use when the low incidence of outcome is taken into consideration. However, LR+ of some CFT (FN, HTS, and RAM) was high, and the clinicians may expect that abnormal findings of the aforementioned have a large effect in increasing the probability of ACI. In the case of ACI or SOL, LR+ of FN, HTS, and RAM were moderate. Other dizziness features—spinning, positional aggravation, or nystagmus—also showed limited diagnostic performance. Instead, extremely low D-dimer value showed the most sensitive power (100%) in the present study. However, a subtle increment of D-dimer level decreased sensitivity substantially. Risk stratification using a blood biomarker is a relatively well-used strategy for acute coronary syndrome or pulmonary embolism among chest pain or dyspnea patients [8–9, 14–15]. Likewise, the researchers expect a clinical role of D-dimer among IVD patients. Further investigation regarding the usability of D-dimer is thus warranted.

In the present study, IVD is defined as dizziness without focal neurologic deficit, except CFT test. This somewhat differs from the traditional definition, because it defined as dizziness without focal neurologic deficit. The traditional definition brings some problems for clinicians. First, the meaning of “without focal neurologic deficit” is ambiguous and sometimes difficult to determine. Senile change or nystagmus can affect the body coordination of fine muscle movement or balance. It is also often difficult to have patients with dizziness or vertigo undergo full neurologic examination, particularly in the case of tandem gait test. Furthermore, a gait test during dizziness can cause a patient to fall down and even become injured. Second, there is no additional description regarding mechanism in the traditional definition. It makes sense that dizziness caused by infection, trauma, or drug reaction should be excluded from the IVD. Therefore, it is suggested that IVD should be defined as the dizziness without any altered mentality, confusion, diplopia, aphasia, dysarthria, cranial nerve palsy including facial palsy, motor or sensory deficit, and without any other mechanisms such as infection, recent trauma, anaphylaxis, drug, and so on.

The study had several limitations. First, the results of the present study are based on patients with IVD and available MRI results, not whole patients with IVD. Some factors could affect the decision for MRI evaluation. It was likely that old patients agreed to an MRI check, but young patients would not. Moreover, socioeconomic status and comorbidities were potential factors, which influence the brain imaging decision. Taking into consideration a bias like this, critical conditions such as already known malignancy and cerebral artery aneurysm were excluded. Of note, the results of the present study should be interpreted regarding the baseline characteristics of enrolled patients.

Second, this study was based on single-center ED data. To date, there is no available data large enough to confirm the prevalence of stroke based on brain images among patients with IVD. Only relatively small and retrospective studies reported the wide range of prevalence. Further studies that enroll bigger populations and settings are needed to achieve generalizability.

Third, the significance of ACI based on MRI sequence may be questionable regarding the clinical impact and medical cost burden. Previously, Savitz et al. described that mortality in cases of misdiagnosed cerebellar infarctions was 40% and half of the survivors retained disabling deficits [2]. However, in the present study, approximately 50% of ACI patients showed subtle infarction size and no significant neurological deficit. Those patients belong to minor stroke [16–17] and, along with TIA, are called transient ischemic attack and minor strokes (TIAMS). Recently, the outpatient or short-stay unit-based treatment strategy has been challenged [18–21].

Fourth, the operator errors should be noted as neurologic evaluation in the present study was performed by junior neurology residents, not senior neurology residents or board-certified physicians. This is the reason why the researcher did not collect data on the type of nystagmus or head impulse test, which requires more experience. However, stroke and peripheral type dizziness can be combined [4], the diagnosis is challenging for skillful physicians without brain images.

Fifth, the association with D-dimer and each of SOL is still remained unknown. Although in the present study, UOR of ACI/SOL was showed statistical significance, we could not proceed the adjusted analysis because of sample missing. Furthermore, D-dimer level was not evaluated yet in frequent conditions among SOL such as meningioma, cerebral aneurysm, or venous anomaly. Thus, it should be cautious at interpreting the statistical result of D-dimer among ACI/SOL.

In conclusion, the present study reports a clinically significant incidence of ACI or SOL among ED patients with IVD. D-dimer showed high sensitive and low specificity, while CFT showed low sensitivity and high specificity.
